# Impact of non-pharmacological interventions on prevention and treatment of delirium in critically ill patients: protocol for a systematic review of quantitative and qualitative research

**DOI:** 10.1186/s13643-016-0254-0

**Published:** 2016-05-04

**Authors:** Leona Bannon, Jennifer McGaughey, Mike Clarke, Daniel Francis McAuley, Bronagh Blackwood

**Affiliations:** Centre for Infection and Immunity, School of Medicine, Dentistry and Biomedical Sciences, Queen’s University Belfast, 97 Lisburn Road, Belfast, BT9 7BL Northern Ireland; School of Nursing and Midwifery, Queen’s University Belfast, 97 Lisburn Road, Belfast, BT9 7BL Northern Ireland; Centre for Public Health, School of Medicine, Dentistry and Biomedical Sciences, Queen’s University Belfast, Belfast, BT12 6BJ Northern Ireland

**Keywords:** Delirium, ICU syndrome, Critical illness, Intensive care unit, Non-pharmacological interventions

## Abstract

**Background:**

Critically ill patients have an increased risk of developing delirium during their intensive care stay. To date, pharmacological interventions have not been shown to be effective for delirium management but non-pharmacological interventions have shown some promise. The aim of this systematic review is to identify effective non-pharmacological interventions for reducing the incidence or the duration of delirium in critically ill patients.

**Methods:**

We will search MEDLINE, EMBASE, CINAHL, Web of Science, AMED, psycINFO and the Cochrane Library. We will include studies of critically ill adults and children. We will include randomised trials and controlled trials which measure the effectiveness of one or more non-pharmacological interventions in reducing incidence or duration of delirium in critically ill patients. We will also include qualitative studies that provide an insight into patients and their families’ experiences of delirium and non-pharmacological interventions. Two independent reviewers will assess studies for eligibility, extract data and appraise quality. We will conduct meta-analyses if possible or present results narratively. Qualitative studies will also be reviewed by two independent reviewers, and a specially designed quality assessment tool incorporating the CASP framework and the POPAY framework will be used to assess quality.

**Discussion:**

Although non-pharmacological interventions have been studied in populations outside of intensive care units and multicomponent interventions have successfully reduced incidence and duration of delirium, no systematic review of non-pharmacological interventions specifically targeting delirium in critically ill patients have been undertaken to date. This systematic review will provide evidence for the development of a multicomponent intervention for delirium management of critically ill patients that can be tested in a subsequent multicentre randomised trial.

**Systematic review registration:**

PROSPERO CRD42015016625

**Electronic supplementary material:**

The online version of this article (doi:10.1186/s13643-016-0254-0) contains supplementary material, which is available to authorized users.

## Background

### Description of the condition

Survivors of critical illness frequently experience ‘malfunction of the cognitive processes in the brain’, known as delirium [1]. The American Psychiatric Association [2] defines delirium as ‘a global disturbance of consciousness characterised by fluctuating mental status, inattention, and disorganised thinking’ which develops over a short period of time and tends to fluctuate throughout the course of the day (p. 127). Delirium is not a disease but a syndrome with a wide spectrum of possible aetiologies [3]. Critically ill patients have an increased risk of developing delirium during their hospital stay. This often results from sepsis and disturbances in inflammation and coagulation pathways leading to microvascular thrombosis [3]. In addition, critical illness disrupts circadian rhythm and sleep patterns and along with sedatives such as benzodiazepines that are commonly used to treat delirium in septic patients, can impair immunity and contribute to delirium [1, 4].

The incidence of delirium is difficult to determine. In the United Kingdom (UK), studies find a 65 % incidence of delirium in mechanically ventilated intensive care unit (ICU) patients [5] and international studies have demonstrated incidence from 25 to 87 % in critically ill patients [6–9]. In a recent point prevalence survey of nine ICUs in the UK, the incidence of delirium was 29 % in adult ICU patients. This study also confirmed that delirium screening practices in the UK remain inconsistent, which may account for the low incidence rates found [10]. Other reasons for a broad range in incidence figures could be differences in incidence in subspecialty ICUs, populations with variable severity of illness and under-recognition of the syndrome [11, 12].

Delirium is potentially modifiable depending on the individual patients’ circumstances. In recent years, the need to introduce validated screening programmes in the ICU has been recognised [13]. In the absence of a valid screening tool, delirium can go unnoticed in up to 70 % of patients [12, 14]. The gold standard for diagnosis of delirium is the DSM-IV criteria applied by a trained psychiatrist, but this method is often not feasible in the hospital setting as psychiatric services are not available around the clock. As a result, multiple delirium detection tools have been developed and validated against DSM-IV criteria for use in the ICU. The Confusion Assessment Method for the Intensive Care Unit (CAM-ICU) and the Intensive Care Delirium Screening Checklist (ICDSC) are the most commonly used tools [6, 15]. The cardinal feature of delirium, inattention [2], is included in both CAM-ICU and ICDSC tools.

Delirium screening and awareness of the associated risk factors are mutually dependent for the successful management of delirium. Van den Boogaard [16] recently developed a delirium prediction model for intensive care patients based on ten risk factors including age, Apache II score, admission group, coma, infection, metabolic acidosis, use of sedatives and morphine, urea concentration and urgent admission. Many of these risk factors are irreversible, but others, such as use of sedatives and morphine, could potentially be modified.

Risk factors for delirium can be divided into three categories, acute illness, host factors including age or chronic health problems and iatrogenic or environmental factors [17]. The iatrogenic or environmental factors include immobilisation, sensory deprivation, sleep deprivation and social isolation [17–24]. Sleep deprivation can be caused by high levels of background noise, absence of natural light, patient care activities, mechanical ventilation, medication, pain, anxiety and stress [25, 26]. These factors have been found to disrupt normal sleep-wake cycles causing sleep deprivation and increasing the risk of delirium [25]. Disrupted sleep in the ICU has been identified as a modifiable precipitating risk factor for delirium [13, 19].

In addition to sleep disruption, mechanically ventilated patients often experience prolonged bed rest and heavy sedation during their ICU stay and this can lead to complications such as ICU weakness or delirium [20]. The introduction of physical and occupational therapy in the earliest days of critical illness has been shown to prevent sedative-related immobility and reduce incidence of delirium [22].

Patients often wake in ICU in unfamiliar surroundings with no recollection of the previous days or even weeks, and this can be extremely confusing for them. Staff need to focus on helping patients adjust to their new environment with regular communication. Education and orientation programmes have shown benefit for delirium management in ICU by increasing awareness of the condition and helping staff cater for patients psychological needs [27]. Targeting these risk factors might help manage the negative outcomes that are associated with delirium.

Patients who experience delirium during critical illness can experience short (during ICU and hospital stay) and long-term (after hospital discharge) sequelae. Studies have shown that short-term outcomes for patients who develop delirium while in ICU are associated with prolonged duration of mechanical ventilation, ICU admission and hospital stay [7, 16, 28, 29]. Evidence suggests that for every extra day patients test positive for delirium, it increases the risk of a prolonged hospital stay by 20 % [8]. Similarly, Salluh et al. [30] in their systematic review and meta-analysis of outcomes of delirium in critically ill patients found that patients with delirium had an increased mean length of stay (approximately 1 day longer) and increased mean duration of mechanical ventilation (almost 2 days longer than patients without delirium), even after adjusting for variables such as age, sex and Apache II scores. Prolonged duration of delirium increases the risk of these negative consequences [31, 32]. Several studies have also identified a link between delirium and in-hospital mortality [5, 12, 16, 26, 28, 33, 34]. These findings suggest that an increased duration of mechanical ventilation, ICU admission and hospital stay often contribute to long-term negative outcomes such as increased mortality and morbidity.

Some critically ill patients have been followed up to 1-year post ICU to study long-term effects of critical illness. These studies found that delirium is an independent risk factor for a threefold higher likelihood of death within 6 months of critical illness even after adjusting for covariates such as severity of illness, coma and use of sedatives [35, 36]. Pisani et al. [23] found that the number of positive delirium days in ICU was significantly associated with time to death in the year following critical illness. Delirium in critical illness can also predict a tenfold higher likelihood of cognitive impairment at 1 year [31]. Studies show that up to six out of every ten patients that survive critical illness struggle with significant cognitive impairment for months to years after their critical illness has resolved. This has significant implications on their quality of life and health care costs and leads to institutionalisation [31]. In addition, delirium is a significant predictor of functional decline and inability to carry out activities of daily living, at both hospital discharge and 3-month follow-up [37]. These negative outcomes incur increased costs and more importantly result in long-term persistent cognitive deficits such as dementia [32, 38] which can significantly impact on a patient’s ability to return to their normal lives.

A longer duration of delirium in ICU is associated with worse outcomes, so removal or reversal of the underlying cause of delirium remains a top priority for successful management of the condition [2]. There has been limited success with pharmacological therapies, results are inconsistent and therapies can be expensive [38]. This lack of evidence on pharmacological therapies for prevention and treatment of delirium is highlighted in the 2013 pain, agitation and delirium (PAD) guidelines of the American College of Critical Care Medicine [13]. Prophylactic antipsychotics and dexmedetomidine have been shown to reduce the prevalence of delirium in critically ill patients. However, no single pharmacologic intervention to prevent or treat delirium has been routinely able to improve mortality rates or hospital length of stay [4, 34, 39–45].

The American College of Critical Care Medicine’s pain, agitation and delirium guidelines recommend non-pharmacological interventions such as early mobilisation [13]. Recently published NICE guidelines [46] recommend a number of non-pharmacological interventions to prevent delirium such as ensuring adequate fluid intake, encouraging exercise or range of motion exercise, introducing cognitively stimulating activities and providing appropriate lighting and clear signage. However, it is worth noting these recommendations are based largely on studies in non-ICU patients (older adults, acute medical) [47–49]. Although this strategy has not been tested on critically ill patients, these non-pharmacological interventions might also benefit ICU patients, but these patients are exposed to many more risk factors for delirium and therefore the proven effectiveness of these interventions in other patients may not be generalizable to them. Therefore, further research into the use of these interventions in an ICU population is needed.

### Description of the intervention

A non-pharmacological intervention is any non-drug intervention. Research in other patient populations is informative for ICU clinicians despite the lack of direct evidence from the ICU setting [49]. Interventions are aimed at targeting risk factors associated with delirium in ICU such as immobilisation, sensory deprivation, sleep deprivation and social isolation and aimed to reduce incidence and/or severity of delirium in critically ill patients. Interventions are not limited to but may include earplugs, eye masks, noise control strategies, pharmacy medication review, music therapy, physical therapy, cognitively stimulating activities, family presence, bright light therapy, education and orientation programmes.

### How the intervention might work

It is hypothesised that a multicomponent non-pharmacological intervention may reduce incidence and severity of delirium by targeting known risk factors such as sensory deprivation, sleep deprivation and immobilisation in critically ill patients. In other patient populations, multicomponent interventions have been successful by targeting modifiable risk factors. For example, Inouye et al., [47] investigated a risk factor targeted protocol for prevention of delirium in an acutely ill elderly population. The protocol targeted sleep deprivation, disorientation, immobility, dehydration and visual and hearing impairments. It successfully achieved a 40 % risk reduction in incidence of delirium in the intervention group. Martinez et al., [48] halved delirium incidence in an acute hospital setting by introducing a delirium prevention protocol delivered by families. Family members were educated about the signs and symptoms of delirium, orientation with familiar objects and photographs and providing hearing aids and eyeglasses. Similarly, Marcantonio et al., [49] achieved an 18 % absolute reduction in the incidence of delirium by introducing a protocol which involved a geriatric consultation early in the surgical hip fracture patient’s admission. The protocol aimed to reduce potentially deliriogenic medications, provide adequate pain relief, control blood pressure, prevent hypoxemia and ensure presence of hearing aids and eyeglasses if needed.

Some single intervention studies of non-pharmacological interventions have also shown promise in the ICU setting, including early mobilisation, earplugs and orientation programmes for patients [22, 26, 27].

### Why is it important to do this review?

Delirium has a high prevalence in ICU and is associated with serious negative outcomes and increased costs. Each delirium incident increases ICU costs 1.3-fold and hospital costs 1.4-fold [38]. Pharmacological interventions are costly and have not shown significant benefit for delirium prevention to date whereas non-pharmacological interventions such as physical therapy, ear plugs and orientation are likely to be less expensive and have shown promise in delirium management in ICU [22, 26, 27]. Additionally, a recently published Cochrane Review on non-pharmacological interventions for sleep promotion in critically ill patients found that the use of earplugs and/or eye masks may have beneficial effects on sleep and delirium incidence but recommended further research due to the poor quality of the studies [50]. There is a growing need and interest in non-pharmacological interventions, and a systematic review of the literature specifically addressing delirium in critically ill patients would help guide ICU staff in delirium management [51, 52]. In addition to providing guidance for staff, the findings would be useful for developing an intervention for future evaluation. This systematic review is both unique and important for two major reasons. First, it will summarise the current evidence and provide an effect estimation for non-pharmacological interventions for delirium targeted at critically ill patients. Secondly, it will synthesise qualitative data from staff, ICU survivors and families’ views on interventions to inform the development and acceptability of non-pharmacological interventions. An understanding of these factors is important for determining the success of the interventions. These interventions are complex, and quantitative studies often do not provide details on factors that can influence the success of the interventions such as workload or implementation process. However, a knowledge of these factors could help ensure the development of a successful intervention.

## Review question

What are the effects of the 11 non-pharmacological interventions named below*, (which could feasibly be delivered within the current roles of the multidisciplinary team in intensive care), on delirium prevention and treatment in Intensive care units?

* (1) earplugs, (2) noise reduction, (3) eye masks, (4) lighting control, (5) education, (6) orientation, (7) cognitive therapy, (8) bright light therapy, (9) music therapy, (10) physical therapy or exercise and (11) pharmacy protocol or review.

## Sub-questions

1. What are ICU survivors and their family members perceptions of non-pharmacological interventions for delirium in ICU?
2. What are clinical staff’s perceptions on acceptability and sustainability of non-pharmacological interventions for delirium in ICU?

## Methods

This protocol was written in accordance to the Preferred Reporting Items for Systematic Reviews and Meta-Analyses Protocols (PRISMA-P) checklist (Additional file 1) [53].

### Types of studies

We will include both quantitative and qualitative studies. The quantitative studies will include clinical trials (randomised trials and other clinical controlled trials including controlled before/after studies and interrupted time series) that evaluate non-pharmacological interventions for reducing incidence and duration of delirium for critically ill patients regardless of age. We will exclude cohort studies, case studies and reports.

The qualitative studies will include studies that use observation, interviews and focus groups that are grounded in phenomenology, ethnography, grounded theory, action research and descriptive research.

### Types of participants

Critically ill patients in the intensive or high dependency unit requiring vasopressors, oxygen therapy, invasive or non-invasive ventilation will be eligible for this review. Both adult and paediatric participants will be included in this review although these will be analysed separately. We will exclude studies of interventions delivered after ICU/high dependency unit (HDU) discharge.

In qualitative studies, the types of participants will be ICU survivors, their families and clinical staff from the ICU environment who have experience caring for patients with delirium.

### Types of intervention(s) and comparators for quantitative studies

We will include studies of non-pharmacological interventions that could be reasonably undertaken by clinicians in the UK NHS, including but not limited to earplugs, noise reduction strategies, eye masks, lighting control, education, orientation, cognitive therapy, bright light therapy, medication review, music therapy and physical therapy. Interventions can include behavioural, cognitive, psychological and physical training interventions. Studies may examine one or a combination of interventions. We will include studies that compare non-pharmacological interventions with either different non-pharmacological interventions, pharmacological interventions (sedatives and antipsychotics) or with no delirium targeted intervention.

We will exclude studies that require trained professionals to deliver the intervention such as acupuncturists or massage therapists that could not reasonably be delivered within the current roles of the multidisciplinary team in ICU.

### Types of outcome measures for quantitative studies

1. Primary outcomes

The primary outcome is the incidence and/or duration of delirium. Delirium must be measured by any delirium screening tool that has been validated against DSM-IV criteria for delirium [2] such as the Confusion Assessment Method for the ICU (CAM-ICU), the Intensive Care Delirium Screening Checklist (ICDSC) or the Neelon and Champagne Confusion Scale (Neecham).

2. Secondary outcomes
  any adverse event reported by the authors
  mortality as reported by the authors
  subjective sleep quality (measures) as reported by participants to authors
  cognitive function (measures) as reported by the authors
  quality of life measured by a validated tool

### Qualitative phenomena of interest

The perception and experiences of ICU survivors, their families and ICU clinical staff are the main qualitative phenomena of interest. As each group has unique perceptions and experiences, the phenomenon of interest will vary. Non-pharmacological interventions are complex, and there are several factors that can influence the success of these interventions in ICU. For clinical staff, their perception of the acceptability and sustainability of non-pharmacological interventions for delirium would include phenomenon related to user friendliness and impact on workload, and their views on how the interventions and process of implementation worked and did not work as applicable are the phenomena of interest. For ICU survivors, the phenomena of interest are memories of non-pharmacological interventions, their views on value and worth of these interventions, views on how they impacted their ICU delirium and views on overall acceptability of these interventions. For families, the phenomena of interest are their degree of involvement with non-pharmacological interventions, i.e. were they involved in education on delirium or orientating their relatives, satisfaction regarding their involvement, their views on how the interventions worked and their satisfaction and views on acceptability and value of the interventions. A deep understanding of these factors is crucial for interpreting the study findings. Quantitative studies do not provide information on how these factors can influence the success of the interventions; however, a knowledge of these factors could help decipher the reasons why an intervention was or was not successful.

### Search strategy

We will search the Cochrane library, MEDLINE (via OVID SP 1966 to present) (Additional file 2), EMBASE (via OVID SP, 1974 to present), CINAHL (via EBSCO, 1981 to present), PsycINFO, (via OVIDSP, 1967 to present), AMED (Allied and Complementary Medicine Database) (via EBSCO host, 1985 to present) and ISI Web of Science (1950 to present). Additional information will be sought from searches of grey literature databases (Opengrey) and NHS databases (NHS Evidence and NICE Guidelines).

Various synonyms for delirium will be searched to include ICU syndrome, acute encephalopathy, cognitive failure, acute brain syndrome, acute confusional state, reversible dementia, ICU psychosis, altered mental state, pseudosenility, toxic encephalopathy, septic encephalopathy, transient organic brain syndrome and acute brain failure. Synonyms for non-pharmacological interventions will include earplugs or ear protective devices, eye masks, relaxation therapy, cognitively stimulating activities, sound masking, orientation programmes, education, bright light therapy, sleep promotion, noise reduction or control, lighting control, therapeutic touch, family presence, exercise or physical therapy, music, behavioural or cognitive therapy, medication review and pharmacological services or protocol or guidelines. Synonyms for critical illness will include critically ill, intensive or critical care and intensive care or critical care unit.

We will attempt to identify ongoing and closed but unpublished studies by searching the major clinical trials registries such as the metaRegister of Controlled Trials (mRCT) (www.isrctn.com/page/mrct), ClinicalTrials.gov (https://clinicaltrials.gov/) and the World Health Organisation (WHO) International Clinical Trials Registry Platform (ICTRP) (http://apps.who.int/trialsearch/). We will also search the reference lists of any identified RCTs and contact primary authors for missing or additional data.

### Selection of studies

One authors (LB) will examine the titles retrieved and exclude any titles that are irrelevant. Two authors (LB, JMcG) will independently examine the abstracts identified from the search. We will retrieve and evaluate the full text of potentially relevant studies. Two authors (LB, JMcG) will independently assess their eligibility according to the review’s eligibility criteria and resolve any disagreements by discussion. A third author (BB) will settle any disagreements. Where appropriate, LB will contact study authors by telephone or email to clarify study eligibility. We will record reasons for study exclusion in the table ‘Characteristics of excluded studies’.

### Data extraction

Data extraction will be independently undertaken by two authors (LB, JMcG).

The data extraction form will be divided into sections to include randomised studies, non-randomised/observational studies and qualitative studies. Data will be extracted from each quantitative study using a data extraction form which will include general study information, study characteristics, participant’s characteristics, intervention and settings, complications and outcome data/results. Data will be extracted from each qualitative study on study setting, population, phenomena of interest, study design, methods, finding and comments.

### Assessment of risk of bias in included quantitative studies

All included studies will be assessed for internal validity and risk of bias using domain-based evaluation as described in the Cochrane Handbook for Systematic Reviews of Interventions, version 5.1.0, chapter 8 [54]. The Cochrane risk of bias form will be used to evaluate each included study. Each study will be assessed as low, high or uncertain risk for the adequacy of sequence generation, allocation concealment, blinding, incomplete outcome data, selective outcome reporting and other bias. Two authors will critically appraise included studies using this tool (LB, JMcG). The Newcastle Ottawa Scale [55] will be used to assess risk of bias in non-randomised studies such as controlled before and after studies and intermittent time series studies.

## Qualitative studies

For qualitative studies, quality assessment will be judged using a tool adapted from the Critical Appraisal Skills Programme (CASP) [56] framework and Popay [57] framework for critical appraisal of qualitative studies and previously used in Jordan et al., [58] (included in Additional file 3). Using this approach, studies were defined as high, moderate or low quality studies as follows; high: criteria was clearly applied and described or communicated from primary author; moderate: criteria not reported clearly and unable to communicate with primary author and finally, low: criteria not applied or applied inappropriately.

## Data analysis—quantitative studies

### Measures of treatment effect

We will use changes in scores from validated delirium screening tools, such as CAM-ICU (Confusion Assessment Method for the Intensive Care Unit), NEElon and CHAMpagne confusion scale (NEECHAM) or ICDSC (Intensive Care Delirium Screening Checklist) to evaluate intervention effect. In the event that change scores are not available, we will use final value data.

### Effect measures for dichotomous outcomes

Where possible, the treatment effect for dichotomous outcomes will be expressed as a risk ratio (RR) with 95 % confidence intervals (CIs).

### Effect measures for continuous outcomes

The treatment effect for continuous outcomes will be displayed as a mean difference with 95 % CI. Where continuous outcomes are measured using difference scales, the treatment effect will be expressed as a standardised mean difference (SMD) with 95 % CI if the results of studies are combined.

### Effect measures for ordinal outcomes and measurement scales

If performing meta-analysis, we will analyse longer ordinal scales as continuous data and we will combine adjacent categories and make them into dichotomous data for shorter ordinal scales. Where ordinal scales are summarised using methods for dichotomous data, we will use risk ratios, odds ratios or risk difference to describe the intervention effect. When ordinal scales are summarised using methods for continuous data, we will calculate mean difference or standardised mean difference to estimate the intervention effect.

### Unit of analysis issues

When alternative measurement scales are used, we will contact the study authors to obtain their participant level data and try to convert the results to standardised units. If a study with more than two intervention groups is included in meta-analysis, we will try to combine relevant groups to create a single pairwise comparison. For cluster randomised trials, we will use the appropriate unit of analysis.

### Dealing with missing data

Wherever possible, we will contact the original authors to request missing data. If more than 20 % of the data are missing from a study, we will exclude the study from the meta-analysis and perform an available case analysis on remaining studies. If possible, we will estimate missing statistics (such as standard error or confidence intervals).

### Assessment of heterogeneity

We will assess heterogeneity of each trial and the intervention effects by compiling ad examining forest plots. We will first explore clinical heterogeneity by assessing clinical and methodological characteristics of the included studies (for example trial design and quality, participant’s characteristics, intervention or outcome measurements). We will only attempt to incorporate data into a meta-analysis if there is negligible clinical heterogeneity among the selected studies.

We will use chi^2^ test (*P* < 0.1, significant heterogeneity) to assess statistical heterogeneity [54]. If the chi^2^ test is significant, we will use the *I*^2^ statistic to measure inconsistency across the studies. *I*^2^ greater than 50 % indicates significant heterogeneity. We will use a fixed-effect model if the studies are homogenous.

### Assessment of reporting biases

We will attempt to obtain and include data from unpublished trials through grey literature searches to reduce the risk of publication bias. We will use funnel plot analysis to assess publication bias when there are greater than ten studies included in the meta-analysis. In interpreting funnel plots, we will explore other reasons for asymmetry such as selection bias, methodological quality and heterogeneity; artefactual and chance.

### Subgroup analysis and investigation of heterogeneity

If sufficient studies are available, we will perform separate analysis on paediatric patients, patients receiving mechanical ventilation versus no mechanical ventilation and studies of interventions aimed at prevention or treatment of delirium.

### Sensitivity analysis

We will test how robust the evidence is by performing sensitivity analysis according to the risk of bias arising from sequence generation and allocation concealment (adequate or unclear or inadequate). We will compare fixed-effect model results to random-effects model results. If necessary, we will undertake sensitivity analysis to examine the effects of excluding study subgroups.

### Summary of findings tables

For quantitative studies, we will use the principles of the GRADE system [59] to assess the quality of the body of evidence associated with specific outcomes reported in the trials. We will include the following outcomes: incidence of delirium, duration of delirium, mortality, quality of life, adverse events and cognitive outcomes in our review and generate a summary of findings (SoF) table using the GRADE software.

## Data synthesis for qualitative studies

A thematic synthesis approach will be undertaken [60]. Coding and thematic development will be conducted manually in three rigorous stages. In the first stage, two reviewers (LB, JMcG) will independently code and arrange the data into themes that involves a continual process of attributing codes to small sections of meaning within the text, moving back and forward across studies and constantly comparing data and codes. In the second stage, the reviewers will work collaboratively, to group codes into logical and meaningful clusters analytical themes relating to the aims of the review will be developed. These will deliver evidence-based recommendations for patient-centred interventions that consider the needs of staff and patients. The third stage will involve assessment of the Confidence in the Evidence from Reviews of Qualitative research (CERQual) [61] which will be used to provide an assessment of confidence for individual review findings from qualitative evidence synthesis; the themes will be summarised in the form of a qualitative findings table. This table is similar to the ‘Summary of findings’ tables used in Cochrane reviews of effectiveness. The four components of the CERQual are the methodological limitations of the qualitative studies contributing to a review finding, the relevance to the review question of the studies contributing to a review finding, the coherence of the review finding and the adequacy of data supporting a review finding. There are four levels for assessment of confidence high, moderate, low and very low [61]. High confidence indicates a high likelihood that the review finding is a reasonable representation of the phenomena of interest, moderate confidence indicates it is likely that the review is a reasonable representation of the phenomenon of interest, low confidence indicates it is possible that the review finding is a reasonable representation of the phenomenon of interest and very low confidence indicates that it is not clear whether the review finding is a reasonable representation of the phenomenon of interest [61].

## Discussion

This systematic review will synthesise research evidence on the effect of non-pharmacological interventions in all critically ill patients. To our knowledge, this synthesis has not been carried out in the ICU population. Non-pharmacological interventions have been studied in populations outside of intensive care units, and multicomponent interventions have successfully reduced incidence and duration of delirium. The NICE guidelines [46] are largely based on studies of non-pharmacological intervention in non-ICU populations [47–49]. Since the publication of these studies, interest in non-pharmacological interventions has increased significantly. This systematic review is important as it provides an update on non-pharmacological interventions for delirium targeted at critically ill patients, and it is also unique as it will provide qualitative data on ICU staff, ICU survivors and their families’ views on interventions to inform the development of the multicomponent bundle.

## Registration

This systematic review has been registered with PROSPERO, an international prospective register of systematic reviews (http://www.crd.york.ac.uk/prospero/display_record.asp?ID=CRD42015016625).

## Additional files

Additional file 1: Preferred Reporting Items for Systematic Review and Meta-Analysis Protocols (PRISMA-P) 2015 checklist. Recommended items to address in a SR protocol. (PDF 147 kb) Additional file 2: MEDLINE search strategy. A list of the keywords used in the MEDLINE search strategy to identify papers for assessment for systematic review. (DOCX 13 kb) Additional file 3: Data extraction form. Data extraction form with section for randomised controlled trials, observational studies and qualitative studies to assess quality and risk of bias. (DOCX 73 kb)

## Abbreviations

CAM-ICU: Confusion Assessment Method for the Intensive Care Unit
CASP: Critical Appraisal Skills Programme
GRADE: Grading of Recommendations Assessment, Development and Evaluation
HDU: high dependency unit
ICDSC: Intensive Care Delirium Screening Checklist
ICU: intensive care unit
PRISMA: Preferred Reporting Items for Systematic Reviews and Meta-Analyses
SoF: summary of findings
UK: United Kingdom

## References

[1] Van Rompaey B, Elseviers MM, Van Drom W, Fromont V, Jorens PG (2012). The effect of earplugs during the night on the onset of delirium and sleep perception: a randomized controlled trial in intensive care patients. Crit Care.

[2] American Psychiatric Association (2000). Diagnostic and statistical manual of mental disorders.

[3] Kress JP (2010). The complex interplay between delirium, sepsis and sedation. Crit Care.

[4] Pandharipande PP, Sanders RD, Girard TD, McGrane S, Thompson JL, Shintani AK (2010). Effect of dexmedetomidine versus lorazepam on outcome in patients with sepsis: an a priori-designed analysis of the MENDS randomized controlled trial. Crit Care.

[5] Page VJ, Navarange S, Gama S, McAuley DF (2009). Routine delirium monitoring in a UK critical care unit. Crit Care.

[6] Ely EW, Margolin R, Francis J, May L, Truman B, Dittus R, Speroff T, Gautam S, Bernard GR, Inouye SK (2001). Evaluation of delirium in critically ill patients: validation of the Confusion Assessment Method for the Intensive Care Unit (CAM-ICU). Crit Care Med.

[7] Mehta S, Cook D, Devlin JW, Skrobik Y, Meade M, Fergusson D (2015). Prevalence, risk factors, and outcomes of delirium in mechanically ventilated adults. Crit Care Med.

[8] Pandharipande P, Cotton BA, Shintani A, Thompson J, Pun BT, Morris JA (2008). Prevalence and risk factors for development of delirium in surgical and trauma intensive care unit patients. J Trauma.

[9] Van den Boogaard M, Schoonhoven L, van der Hoeven JG, van Achterberg T, Pickkers P (2012). Incidence and short-term consequences of delirium in critically ill patients: a prospective observational cohort study. Int J Nurs Stud.

[10] Giraud K, Vuylsteke A (2014). Point-prevalence of delirium in intensive care units. Anaesthesia.

[11] Faria S, Moreno RP (2013). Delirium in intensive care: an under-diagnosed reality. Rev Bras Ter Intensiva.

[12] Spronk PE, Riekerk B, Hofhuis J, Rommes JH (2009). Occurrence of delirium is severely underestimated in the ICU during daily care. Intensive Care Med.

[13] Barr J, Fraser GL, Puntillo K, Ely EW, Gelinas C, Dasta JF (2013). Clinical practice guidelines for the management of pain, agitation, and delirium in adult patients in the intensive care unit. Crit Care Med.

[14] Van Eijk MM, van Marum RJ, Klijn IA, de Wit N, Kesecioglu J, Slooter AJ (2009). Comparison of delirium assessment tools in a mixed intensive care unit. Crit Care Med.

[15] Bergeron N, Dubois MJ, Dumont M, Dial S, Skrobik Y (2001). Intensive Care Delirium Screening Checklist: evaluation of a new screening tool. Intensive Care Med.

[16] Van den Boogaard M, Pickkers P, Slooter AJC, Kuiper MA, Spronk PE, van der Voort PHJ (2012). Development and validation of PRE-DELIRIC (PREdiction of DELIRium in ICu patients) delirium prediction model for intensive care patients: observational multicentre study. BMC.

[17] Ely EW, Siegel MD, Inouye SK (2001). Delirium in the intensive care unit: an under-recognized syndrome of organ dysfunction. Semin Respir Crit Care Med.

[18] Larkin M, Wilson MD (1972). Intensive care delirium: the windowless unit. Arch Int Med.

[19] Kamdar BB, Needham DM, Collop NA (2012). Sleep deprivation in critical illness: its role in physical and psychological recovery. J Intensive Care Med.

[20] Needham DM (2008). Mobilizing patients in the intensive care unit: improving neuromuscular weakness and physical function. JAMA.

[21] Needham DM, Korupolu R, Zanni JM, Pradhan P, Colantuoni E, Palmer JB (2010). Early physical medicine and rehabilitation for patients with acute respiratory failure: a quality improvement project. Arch Phys Med Rehabil.

[22] Schweickert WD, Pohlman MC, Pohlman AS, Nigos C, Pawlik AJ, Esbrook CL (2009). Early physical and occupational therapy in mechanically ventilated, critically ill patients: a randomised controlled trial. Lancet.

[23] Pisani MA, Kong SY, Kasl SV, Murphy TE, Araujo KL, Van Ness PH (2009). Days of delirium are associated with 1-year mortality in an older intensive care unit population. Am J Respir Crit Care Med.

[24] Ouimet S, Kavanagh BP, Gottfried SB, Skrobik Y (2007). Incidence, risk factors and consequences of ICU delirium. Intensive Care Med.

[25] Gabor JY, Cooper AB, Crombach SA, Lee B, Kadikar N, Bettger HE (2003). Contribution of the intensive care unit environment to sleep disruption in mechanically ventilated patients and healthy subjects. Am J Respir Crit Care Med.

[26] Van Rompaey B, Elseviers M, Bossaert L (2009). Risk factors for intensive care delirium: a prospective cohort study. Crit Care.

[27] Colombo R, Corona A, Praga F, Minari C, Giannotti C, Castelli A (2012). A reorientation strategy for reducing delirium in the critically ill. Results of an interventional study. Minerva Anestesiol.

[28] Shehabi Y, Riker RR, Bokesch PM, Wisemandle W, Shintani A, Ely EW (2010). Delirium duration and mortality in lightly sedated, mechanically ventilated intensive care patients. Crit Care Med.

[29] Van den Boogaard M, Schoonhoven L, Evers AW, van der Hoeven JG, van Achterberg T, Pickkers P (2012). Delirium in critically ill patients: impact on long-term health-related quality of life and cognitive functioning. Crit Care Med.

[30] Salluh JI, Wang H, Schneider EB, Nagaraja N, Yenokyan G, Damluji A (2015). Outcome of delirium in critically ill patients: systematic review and meta-analysis. BMJ.

[31] Girard TD, Jackson JC, Pandharipande PP, Pun BT, Thompson JL, Shintani AK (2010). Delirium as a predictor of long-term cognitive impairment in survivors of critical illness. Crit Care Med.

[32] Pandharipande PP, Girard TD, Jackson JC, Morandi A, Thompson JL, Pun BT (2013). Long-term cognitive impairment after critical illness. N Engl J Med.

[33] Lin SM, Liu CY, Wang CH, Lin HC, Huang CD, Huang PY (2004). The impact of delirium on the survival of mechanically ventilated patients. Crit Care Med.

[34] Van den Boogaard M, Schoonhoven L, van Achterberg T, van der Hoeven JG, Pickkers P (2013). Haloperidol prophylaxis in critically ill patients with a high risk for delirium. Crit Care.

[35] Ely EW, Shintani A, Truman B, Speroff T, Gordon SM, Harrell FE (2004). Delirium as a predictor of mortality in mechanically ventilated patients in the intensive care unit. JAMA.

[36] Van Rompaey B, Schuurmans MJ, Shortridge-Baggett LM, Truijen S, Bossaert L (2008). Risk factors for delirium in intensive care patients: a systematic review. Intensive Crit Care Nurs.

[37] Inouye SK, Rushing JT, Foreman MD, Palmer RM, Pompei P (1998). Does delirium contribute to poor hospital outcomes? A three-site epidemiologic study. J Gen Intern Med.

[38] Milbrandt EB, Deppen S, Harrison PL, Shintani AK, Speroff T, Stiles RA (2004). Costs associated with delirium in mechanically ventilated patients. Crit Care Med.

[39] Maldonado JR, Wysong A, van der Starre PJ, Block T, Miller C, Reitz BA (2009). Dexmedetomidine and the reduction of postoperative delirium after cardiac surgery. Psychosomatics.

[40] Riker RR, Shehabi Y, Bokesch PM, Ceraso D, Wisemandle W, Koura F (2009). Dexmedetomidine vs midazolam for sedation of critically ill patients: a randomized trial. JAMA.

[41] Wang HR, Woo YS, Bahk WM (2013). Atypical antipsychotics in the treatment of delirium. Psychiatry Clin Neurosci.

[42] Hakim SM, Othman AI, Naoum DO (2012). Early treatment with risperidone for subsyndromal delirium after on-pump cardiac surgery in the elderly: a randomized trial. Anesthesiology.

[43] Prakanrattana U, Prapaitrakool S (2007). Efficacy of risperidone for prevention of postoperative delirium in cardiac surgery. Anaesth Intensive Care.

[44] Mardani D, Bigdelian H (2013). Prophylaxis of dexamethasone protects patients from further post-operative delirium after cardiac surgery: a randomized trial. J Res Med Sci.

[45] Page VJ, Ely EW, Gates S, Zhao XB, Alce T, Shintani A (2013). Effect of intravenous haloperidol on the duration of delirium and coma in critically ill patients (Hope-ICU): a randomised, double-blind, placebo-controlled trial. Lancet Respir Med.

[46] National Institute for Health and Care Excellence (2014). Delirium: quality statement two: interventions to prevent delirium, QS63.

[47] Inouye SK, Bogardus ST, Charpentier PA, Leo-Summers L, Acampora D, Holford TR (1999). A multicomponent intervention to prevent delirium in hospitalized older patients. N Engl J Med.

[48] Martinez FT, Tobar C, Beddings CI, Vallejo G, Fuentes P (2012). Preventing delirium in an acute hospital using a non-pharmacological intervention. Age Ageing.

[49] Marcantonio ER, Flacker JM, Wright RJ, Resnick NM (2001). Reducing delirium after hip fracture: a randomized trial. J Am Geriatr Soc.

[50] Hu RF, Jiang XY, Chen J, Zeng Z, Chen XY, Li Y, Huining X, Evans DJ (2015). Non-pharmacological interventions for sleep promotion in the intensive care unit. Cochrane Database Syst Rev.

[51] Tabet N, Howard R (2009). Non-pharmacological interventions in the prevention of delirium. Age Ageing.

[52] Zaal IJ, Spruyt CF, Peelen LM, van Eijk MM, Wientjes R, Schneider MM (2013). Intensive care unit environment may affect the course of delirium. Intensive Care Med.

[53] Moher D, Shamseer L, Clarke M, Ghersi D, Liberati A, Petticrew M, Shekelle P, Stewart LA (2015). Preferred reporting items for systematic review and meta-analysis protocols (PRISMA-P) 2015 statement. Syst Rev.

[54] Higgins JPT, Green S, editors. The Cochrane Handbook for Systematic Reviews of Interventions version 5.1.0, updated March 2011. The Cochrane Collaboration, 2011. http://handbook.cochrane.org/. Accessed 10 Aug 2015.

[55] Newcastle Ottawa Wells G, Shea B, O'Connell J, Robertson J, et al. The Newcastle-Ottawa Scale (NOS) for assessing the quality of nonrandomised studies in meta-analysis. 2011. [Website]. http://www.ohri.ca/programs/clinical_epidemiology/nosgen.pdf. Available from: URL: http://www.ohri.ca/programs/clinical_epidemiology/nosgen.pdf. Accessed 12 Nov 2015.

[56] Critical Appraisal Skills Programme (CASP) 2014. CASP Checklists Available from URL: (http://media.wix.com/ugd/dded87_29c5b002d99342f788c670e49f274.pdf) Oxford. CASP (Accessed 02 Dec 15).

[57] Popay J, Rogers A, Williams G (1998). Rationale and standards for the systematic review of qualitative literature in health services research. Qual Health Res.

[58] Jordan J, Rose L, Noyes J, Dainty KN, Blackwood B. Factors that impact on protocolized weaning from mechanical ventilation in critically ill adults & children: a Cochrane qualitative synthesis (Protocol). Cochrane Database of Systematic Reviews Issue 5, 16 May. 2012. 10.1002/14651858.CD011812.pub2 PMC6458040 27699783

[59] Thomas J, Harden A (2008). Methods for the thematic synthesis of qualitative research in systematic reviews. BMC Med Res Methodol.

[60] Atkins D, Eccles M, Flottorp S, Guyatt G, Henry D, Hill S, Liberati A, O'Connell D, Oxman AD, Phillips B, Schünemann H, Tan-Torres Edejer T, Vist GE, Williams J, The GRADE Working Group (2004). Grading quality of evidence and strength of recommendations. BMJ.

[61] Lewin S, Glenton C, Munthe-Kaas H, Carlsen B, Colvin CJ, Gulmezogu M (2015). Using qualitative evidence in decision making for health and social interventions: an approach to assess confidence in findings from qualitative evidence synthesis (GRADE-CERQual). PLoS Med.

