# Supplementary material for: Impact of non-pharmacological interventions on prevention and treatment of delirium in critically ill patients: protocol for a systematic review of quantitative and qualitative research
Source: Syst Rev. 2016 May 4;5:75. doi: 10.1186/s13643-016-0254-0 (PMC4855765; doi:10.1186/s13643-016-0254-0)
Supplement: Additional file 2: — MEDLINE search strategy. A list of the keywords used in the MEDLINE search strategy to identify papers for assessment for systematic review. (DOCX 13 kb) [file 13643_2016_254_MOESM2_ESM.docx]

Search strategy- MEDLINE

1. Delirium
2. ICU syndrome
3. Cognitive failure
4. Acute brain syndrome
5. Acute confusional state
6. Reversible dementia
7. ICU psychosis
8. Altered mental state
9. Pseudosenility
10. Toxic encephalopathy
11. Septic encephalopathy
12. Transient organic brain syndrome
13. Acute brain failure
14. 1 or 2 or 3 or 4 or 5 or 6 or 7 or 8 or 9 or 10 or 11 or 12 or 13
15. Critically ill patients
16. Critical* and ill*
17. Intensive care
18. Critical care
19. Intensive or critical and unit*
20. 15 or 16 or 17 or 18 or 19
21. Earplugs or ear protective devices
22. Eyemasks
23. Relaxation
24. Cogni*
25. Sound masking
26. Orientat*
27. Education or Educat*
28. Bright light therapy
29. Sleep and (promot* or help* or support* or initiat*)
30. Noise and (reduct* or control)
31. Lighting and (reduct* or control)
32. Therapeutic touch
33. Famil*
34. Sedat*
35. Exercise*
36. Music or complementary or alternative or cognitive or behavioural or physical (and therap*)
37. Pharmac* services or review or protocol
38. 21 or 22 or 23 or 24 or 25 or 26 or 27 or 28 or 29 or 30 or 31 or 32 or 33 or 34 or 35 or 36 or 37
39. 14 and 20 and 38
