# Supplementary material for: Impact of non-pharmacological interventions on prevention and treatment of delirium in critically ill patients: protocol for a systematic review of quantitative and qualitative research
Source: Syst Rev. 2016 May 4;5:75. doi: 10.1186/s13643-016-0254-0 (PMC4855765; doi:10.1186/s13643-016-0254-0)
Supplement: Additional file 3: — Data extraction form. Data extraction form with section for randomised controlled trials, observational studies and qualitative studies to assess quality and risk of bias. (DOCX 73 kb) [file 13643_2016_254_MOESM3_ESM.docx]

**Study selection, Quality Assessment & Data Extraction Form**

Name of author extracting data: _____________________________________

Date form completed: _____________________________________

**Study ID**

| Title |  |
| --- | --- |
| Study ID  (Family name of first author and year of publication and letter if more than one year, e.g. Bannon2015a) |  |
| Are there other articles of same?  (Yes, No, Unclear. If Yes, write study IDs) |  |

**Study Eligibility**

|  | **(please circle)** |  |
| --- | --- | --- |
| **Type of studies**  1. Can this study be considered a randomised controlled trial or controlled trial including controlled before and after and intermittent time series? or  **3.** Is this a qualitative study? (please complete qualitative section below) | **Yes, Unclear, No** |  |
| **Types of participants**  1. Were the participants’ critically ill patients (defined as patients cared for in critical care and intubated and ventilated or receiving oxygen therapy or vasopressors) or relatives of critically ill patients or clinical staff caring for critically ill patients? | **Yes, Unclear, No** |  |
| **Interventions**  Can the intervention be considered non-pharmacological and can it be delivered within the current roles of the usual multidisciplinary team in ICU without the need for specialist staff? Or  Is this a qualitative study that examines lived experiences of delirium or an intervention? | **Yes, Unclear, No** |  |
| **Outcomes:**  Did the study report?  1. Incidence of delirium or  2. Duration of delirium or  3. Qualitative insights into non-pharmacological intervention for delirium management in the critically ill | **Yes, Unclear, No** |  |
| **Conclusion:**  Do not proceed if any of the previous answers are ‘No’.   - **Included** - **Excluded and listed in excluded table** - **More information needed before inclusion decision (specify)** | | |

**Please complete the next section for randomised trials, please see below for observational or qualitative research sections.**

***Randomised controlled studies***

| **PARTICIPANTS** | | | | | | | | |
| --- | --- | --- | --- | --- | --- | --- | --- | --- |
| **Inclusion/Exclusion Criteria** | | | | | | | | |
|  | | | | | | | | |
|  | | **Intervention** |  | | | | **Control** | |
| **Number randomised** | |  |  | | | |  | |
| **Number analyzed** | |  |  | | | |  | |
| **Age, mean (SD)**  **Median (IQR)** | |  | **Age, mean (SD)**  **Median (IQR)** | | | |  | |
| **Male n (%)**  **Female n (%)** | |  | **Male n (%)**  **Female n (%)** | | | |  | |
| **Delirium screening tool used** | |  |  | | | |  | |
| **Setting** | **Participating site country (ies):** | | | |  | | |  |
| - **Academic hospital** | | | | - **Non-teaching hospital** | | - **Not reported** | |  |
| **Number of ICUs and types (e.g. medical; surgical; mixed; neuro. Include no of beds if reported) include if paediatric or adult patients, include info re: ventilation, vasopressors or o2 therapy** | |  | | | | | |  |
| **Intervention** | | | | | | | |  |
| **Describe intervention, who delivered it? How was it delivered? Duration of treatment? How many times per day?** | | | | | | | |  |
| **Comparator**  **Describe standard care for delirium/non-pharmacological intervention studied as comparator** | | | | | | | |  |
| **Outcomes** | | | | | | | |  |
| **Primary** | | \|  \| **Incidence of delirium** \| - **Not measured** - **Yes** - **No** - **Unclear** \| \| --- \| --- \| --- \| \|  \| **Duration of delirium** \| - **Not measured** - **Yes** - **No** - **Unclear** \| \|  \| **No of delirium cases treated pharmacologically** \| - **Not measured** - **Yes** - **No** - **Unclear** \| | | | | | |  |
| **Secondary** | | \|  \| **Any adverse events reported by original authors** \| - **Not measured** - **Yes** - **No** - **Unclear** \| \| --- \| --- \| --- \| \|  \| **Mortality reported by the authors** \| - **Not measured** - **Yes** - **No** - **Unclear** \| \|  \| **Subjective sleep Quality as reported by participants;** \| - **Not measured** - **Yes** - **No** - **Unclear** \| \|  \| **Cognitive function as reported by the original authors;** \| - **Not measured** - **Yes** - **No** - **Unclear** \| \|  \| **Quality of life measured by a validated tool** \| - **Not measured** - **Yes** - **No** - **Unclear** \| | | | | | |  |

| **Domain** | **Description** | **Judgement** |
| --- | --- | --- |
| **Sequence generation**  **Was the allocation sequence adequately generated?** |  | - **Yes** - **No** - **Unclear** |
| **Allocation concealment**  **Was allocation adequately concealed?** |  | - **Yes** - **No** - **Unclear** |
| **Blinding (participants, personnel, outcome)**  **Was knowledge of the allocation intervention adequately prevented during the study?** |  | - **Yes** - **No** - **Unclear** |
| **Selective outcome reporting. Are reports of the study free of suggestion of selective outcome reporting?** |  | - **Yes** - **No** - **Unclear** |
| **Other sources of bias.**  **Study free from other bias?** |  | - **Yes** - **No** - **Unclear** |

**Please complete the next section for observational studies**

***Observational studies***

| Intervention | Intervention type |
| --- | --- |
| - Preventative - Therapeutic | - Earplugs or noise reduction, - Eye masks or lighting control, - Education, - Orientation, - Cognitive therapy, - Bright light therapy, - Music therapy, - Physical therapy or exercise - Pharmacy protocol or review. - Other (please specify) |

| What Observational Study (OS) design was used? |
| --- |
| - Case control      - Interrupted time series - Before-after/ pre-post - Cross-sectional |
| **Only include case control, controlled before after/ pre-post and interrupted time series design in this review as defined a priori in protocol.** |

| **PARTICIPANTS** | | | | | | | | |
| --- | --- | --- | --- | --- | --- | --- | --- | --- |
| **Inclusion/Exclusion Criteria** | | | | | | | | |
|  | | | | | | | | |
|  | | **Pre-intervention** | |  | | | **Post-intervention** | |
| **Number** | |  | |  | | |  | |
| **Number analyzed** | |  | |  | | |  | |
| **Age, mean (SD)**  **Median (IQR)** | |  | | **Age, mean (SD)**  **Median (IQR)** | | |  | |
| **Male n (%)**  **Female (%)** | |  | | **Male n (%)**  **Female (%)** | | |  | |
| **Delirium screening tool used** | |  | |  | | |  | |
| **Setting** | **Participating site country (ies):** | | | |  | | |  |
| - **Academic hospital** | | | - **Non-teaching hospital** | | | - **Not reported** | |  |
| **Number of ICUs and types (e.g. medical; surgical; mixed; neuro. Include no of beds if reported)** | |  | | | | | |  |
| **Intervention** | | | | | | | |  |
| **Describe intervention, who delivered it? How was it delivered? Duration of treatment? How many times per day?** | | | | | | | |  |
| **Comparator**  **Describe standard care for delirium/non-pharmacological intervention used as comparator** | | | | | | | |  |

**Study results**

| Outcome | Intervention ( )  number/ total number | Control/ comparator( )  number / total number |
| --- | --- | --- |
| Incidence of delirium |  |  |
| Duration of delirium |  |  |
| No of cases treated pharmacologically |  |  |
| Adverse events reported by authors |  |  |
| In hospital mortality |  |  |
| Hospital mortality |  |  |
| Subjective sleep quality reported by patients |  |  |
| Cognitive function at discharge |  |  |
| Quality of life at discharge from hospital |  |  |

**Assessment of bias**

**[Newcastle Ottawa Scale, 2011] {54}**

| **Selection**  1) Is the case definition adequate?  a) yes, with independent validation  b) yes, eg record linkage or based on self reports  c) no description |  |
| --- | --- |
| 2) Representativeness of the cases  a) consecutive or obviously representative series of cases  b) potential for selection biases or not stated |  |
| 3) Selection of Controls  a) community controls  b) hospital controls  c) no description |  |
| 4) Definition of Controls  a) no history of disease (endpoint)  b) no description of source |  |
| **Comparability**  1) Comparability of cases and controls on the basis of the design or analysis  a) study controls for _______________ (Select the most important factor.)  b) study controls for any additional factor  ___________________  ____________________  ____________________ |  |
| **Exposure**  1) Ascertainment of exposure  a) secure record (eg surgical records)  b) structured interview where blind to case/control status  c) interview not blinded to case/control status  d) written self report or medical record only  e) no description |  |
| 2) Same method of ascertainment for cases and controls  a) yes  b) no |  |
| 3) Non-Response rate  a) same rate for both groups  b) non respondents described  c) rate different and no designation |  |

**Please leave the next section blank if the study does not contain any qualitative data.**

***Qualitative studies***

| Was the study conducted as… | - Stand-alone qualitative study - Part of a larger qualitative study - Part of larger mixed methods study | | |
| --- | --- | --- | --- |
| Focus of study | - Implementation of a non-pharmacological intervention - Evaluation of a non-pharmacological intervention - Mixed (please specify) - Other (please specify) | | |
| Aims & Objectives |  | | |
| Phenomena of interest e.g. attitudes / perceptions / knowledge / understandings / behaviour |  | | |
| **Study population:** | | ICU staff | ICU Survivors & family |
|  | | Years experience ______  Grade ______  Gender ______  Number ______ | - Patient - Family member   Duration of ICU stay _______  Numbers _______ |
| Method of selection e.g purposive/convenience sampling | |  |  |
| Inclusion/Exclusion criteria | |  |  |

**Theoretical/ conceptual framework**

| Stated framework/orientation | - Phenomenology - Grounded theory - Descriptive research - Ethnography - Action research - Other (please specify) ___________   ____________________________ |
| --- | --- |
| Detail provided re: chosen framework |  |

**Data collection**

| Method of data collection | - Unstructured - Semi-structured individual interviews - Focus groups - Participant observation - Non-participant observation - Other (please specify) ____________   ______________________________ |
| --- | --- |
| Method of data recording | - Written - Digital recording - Video recording - Other (please specify) ____________   _____________________________ |
| Detail provide re: chosen data collection methods e.g. rationale for choice of methods/ how these methods relate to theoretical/conceptual framework |  |

**Data analysis**

| Method of data analysis | - Thematic content analysis - Grounded theory - Discourse analysis - Narrative analysis - Other (please specify) ___________   ____________________________ |
| --- | --- |
| Procedures for data analysis e.g. use of computer software package, process(es) of coding |  |
| Details provided re: chosen data analysis method |  |

**Research rigour**

**Quality Appraisal Checklist**

(see Jordan, 2012) {59}

| **Quality Appraisal Questions** | **Study reference** | **Decision (either ‘Yes’, ‘No’ or ‘Unclear’)** |
| --- | --- | --- |
| 1. Was there a clear statement of the aims of the research? |  |  |
| 2. Is a qualitative methodology appropriate? |  |  |
| 3. Was the research design process appropriate to address the aims of the research? |  |  |
| 4. Was the recruitment strategy appropriate to the aims of the research? |  |  |
| 5. Is there a clear and detailed statement of findings? (How ‘rich’ are the findings in terms of their detail and conceptual / theoretical development?) |  |  |
| 6. Is there clear evidence of steps taken to enhance the validity and reliability of study process / findings? |  |  |
| 7. Is there evidence of consideration of the risk of bias in the production of findings (i.e. from a qualitative position - a reflexive concern with the researcher’s standpoint)? |  |  |
| 8. Is there evidence of analysis and interpretation of the findings at a conceptual and theoretical level? |  |  |
| 9. Is there evidence of consideration of the generalisability of study findings, that is, how they are related to broader theoretical concerns and/or other empirical contexts? |  |  |

| **Phenomena of interest: ICU survivors, their families and ICU clinical staffs’** |
| --- |

| **ICU Clinical staff** |  |
| --- | --- |
| User friendliness of the intervention |  |
| Impact on workload |  |
| Views on how the interventions worked or didn’t work (as applicable) |  |
| Views on how the process of implementation worked or didn’t work (as applicable) |  |
| **ICU survivors** |  |
| Memories of non-pharmacological interventions in ICU (if any) |  |
| Views on value or worth of non-pharmacological interventions in ICU |  |
| Views on how non-pharmacological interventions impacted delirium in ICU |  |
| Views on overall acceptability of non-pharmacological interventions in ICU |  |
| **ICU survivors families** |  |
| Degree of involvement with non-pharmacological intervention in ICU |  |
| Satisfaction with degree of involvement |  |
| Views on how the interventions worked or didn’t work (as applicable) |  |
| Overall acceptability and views on value of non-pharmacological interventions in ICU |  |

| Author(s) inferences/ implications for practice/policy |  |
| --- | --- |
| Author(s) conclusions |  |
| Any other issues/ comments |  |
